# Genome-Wide Association Study in Vestibular Neuritis: Involvement of the Host Factor for HSV-1 Replication

**DOI:** 10.3389/fneur.2018.00591

**Published:** 2018-07-20

**Authors:** Dan Rujescu, Annette M. Hartmann, Ina Giegling, Bettina Konte, Marko Herrling, Susanne Himmelein, Michael Strupp

**Affiliations:** ^1^German Center for Vertigo and Balance Disorders, University Hospital Munich, Munich, Germany; ^2^Department of Psychiatry, Psychotherapy and Psychosomatics, Martin-Luther-University Halle-Wittenberg, Halle, Germany; ^3^Department of Neurology, University Hospital Munich, Munich, Germany

**Keywords:** vestibular neuritis, Herpes simplex virus type 1, genome-wide association study, virus infection, insulin metabolism

## Abstract

**Objective:** In order to identify genetic variants associated with vestibular neuritis, a common cause of peripheral vertigo with a potential causative link to the reactivation of herpes simplex type 1 (HSV-1), we conducted a genome-wide association study.

**Methods:** Association was assessed using approximately 8 million variants. 131 patients with vestibular neuritis and 2,609 controls of European ancestry were included.

**Results:** Genome-wide associations with vestibular neuritis were detected in 4 regions containing protein coding genes assignable to two functional groups: virus hypothesis and insulin metabolism. Genes of set 1 are related to viral processes: nuclear receptor subfamily 3 group C member 2 (NR3C2) is a receptor for mineralocorticoids and glucocorticoids and was shown to be a host factor for HSV-1 replication. Ankyrin repeat domain 30A (ANKRD30A) encodes a host factor for human immunodeficiency virus-1 (HIV-1) infection. It shows rapid evolution and is induced by interferon stimulation. Mediator complex 30 (MED30), an important member of the mediator complex, has been shown to be involved in replication of HIV-1, a knockdown leading to impaired viral replication. The second set of genes LIM homeobox transcription factor 1 alpha (LMX1A), solute carrier family 30 member 8 (SLC30A8) is associated with insulin metabolism and resistance, a feature of some patients in whom type 2 diabetes is an accompanying comorbidity of vestibular neuritis.

**Conclusions:** Using a GWAS approach to evaluate the etiology of vestibular neuritis these findings provide another piece of evidence that it may be caused by a viral inflammation.

## Introduction

Evidence for a genetic background of episodic or progressive vestibular disorders is beginning to emerge [reviewed in (1, 2)]. Common variants are revealed through genome-wide association studies [e.g., motion sickness, (3)], and rare variants through linkage analyses followed by whole exome sequencing (e.g., familial Meniere's disease) (4, 5). For vestibular neuritis (VN), the third most common cause of peripheral vestibular vertigo, evidence for heritability is very low and linkage studies have not been published yet.

The key symptoms of VN, also known as acute unilateral vestibulopathy, are acute onset of sustained spinning vertigo, oscillopsia, postural imbalance and nausea and vomiting lasting for many days or a few weeks (6). An annual incidence of 15.5 per 100,000 persons was described (7, 8). Age of onset is typically between 30 and 60 years (7), with an accumulation between 40 and 50 years (7, 8). Reported recurrence rates vary between 1.9% (9) and 10.7% (10).

The most popular hypothesis of viral etiology is based on several supporting observations, including (1) a profound similarity of the vestibular nerve histopathology between cases of VN and singular cases of herpes zoster oticus, (2) the deployment of herpes simplex type 1 (HSV-1) to establish an animal model of VN by inoculation of the virus into the auricle of mice, (3) detection of the latency associated transcript of HSV-1 in about 2/3 of autopsied human vestibular ganglia by PCR (11). These findings indicate that HSV-1 resides in a latent state in the vestibular ganglia. Reactivation of HSV-1 caused by inter-current factors then leads to sudden replication and induction of inflammation, edema and secondary cell damage of the vestibular ganglion cells and axons in the bony canals [reviewed in (6)].

One of the main comorbidities in patients with VN is type 2 diabetes mellitus (T2DM) (7), which is characterized by insulin resistance and an imbalance in glucose homoeostasis. It has been suggested that the load of viral infection associated with chronic low-grade inflammation might result in insulin resistance (12).

In the current approach we used a genome-wide association study to identify genes which might be related to the disease in order to further evaluate the etiology of VN.

## Subjects/materials and methods

We performed a GWAS with patients with VN of European ancestry, collected by the German Center for Vertigo and Balance disorders (Munich, Germany), and healthy volunteers of German descent, randomly selected from the general population of Munich.

### Patients

Patients of Caucasian descent were recruited in the German Center for Vertigo and Balance Disorders in Munich, Germany from 2012 to 2016. Detailed medical histories of the participants and their first-degree relatives were assessed using a structured interview.

#### Inclusion criteria

All patients to be included had to fulfill the diagnostic criteria for unilateral VN (6). The diagnosis was defined by the patient history, the clinical examination and, in ambiguous cases, laboratory examinations. Patients with recurrent VN were not present. The inclusion criteria for this study were as follows:

##### Key symptoms

History of an acute/subacute onset of sustained spinning vertigo; apparent movement of the visual surroundings (oscillopsia), gait and postural imbalance with a tendency to fall toward the side of the affected ear; nausea or vomiting. All symptoms had to last for at least 72 h.

##### Key signs

Horizontal-rotatory peripheral vestibular spontaneous nystagmus toward the non-affected ear, pathological head-impulse test (HIT) toward the affected side, postural imbalance with Romberg fall toward the affected ear.

##### Laboratory examinations

Caloric testing showed a hypo- or unresponsiveness of the tested and affected horizontal canal with an >25% asymmetry between the two sides; if the bedside head impulse was not clearly pathological the video-HIT had to be performed with a gain of the vestibulo-ocular reflex <0.7 on the affected side).

#### Exclusion criteria

(1) Any evidence for other vestibular disorders, such as Menière's disease, vestibular paroxysmia, bilateral vestibulopathy, vestibular migraine or brainstem/cerebellar infarction. (2) History of acute hearing loss or brainstem or cerebellar symptoms associated with these symptoms. (3) Clinical evidence for a central lesion, i.e., skew-deviation, saccadic smooth pursuit, gaze-evoked nystagmus, normal head-impulse test or other central signs.

### Healthy volunteers (PAGES)

As part of the PAGES (Phenomics and Genomics Sample) sample approximately 3,000 inhabitants from the greater Munich area were randomly selected. Healthy unrelated volunteers of German ancestry were included. A semi-structured interview was used to assess a detailed medical, neurological and psychiatric history of the participants, including information on their first-degree relatives, and was followed by a comprehensive interview including the Structured Clinical Interview for DSM-IV (SCID I and SCID II) (13, 14), and the Family History Assessment Module (15). CNS impairment was ruled out using an orienting neurological examination. Subjects with a self-reported history of VN were excluded. Finally, individuals suffering from neurological or psychiatric diseases were excluded.

### Participant consent

Informed consent was obtained from all participants. The study was approved by the ethics committee of the Ludwig-Maximilians-University Munich and carried out in accordance with the Declarations of Helsinki.

### DNA extraction

Genomic DNA was extracted from whole blood using the QIAamp DNA Maxi Kit (Qiagen), according to the manufacturer's instructions and dissolved in nuclease free water. The concentration of genomic DNA was measured using picogreen (Molecular Probes) and adjusted to 50 ng/μl.

### Genome-wide association analysis

#### Overview

Samples were genotyped on different platforms, imputed in seven batches and combined into one large dataset. Quality control and imputation of batch 1 [Human610-Quad (16), Human660W-Quad (17)], batch 2 [HumanHap 300 (18)] and 3 [Affymetrix 6.0 (19)] were performed in the framework of a schizophrenia meta-analysis conducted by the Psychiatric Genomics Consortium (PGC) (20). Batches 4 [HumanHap 300 (21)], 5 [Illumina Human OmniExpress 12 (22)], 6 [Illumina Omni1-Quad (23)], and 7 (HumanOmniExpress-24) were processed following quality control and imputation protocols used by the PGC. Datasets were combined to get a sufficient number of controls. Patients in batch 7 were recruited for studying vertigo and balance disorders, whereas patients of batches 1–6 were recruited for studying psychiatric diseases, mainly schizophrenia, and thus not included in the conducted genome-wide analysis (see Table 1). Appropriate cases and controls were selected after global quality control.

**Table 1:** Batch distribution of cases and controls.

	**Post-imputation**	**GWAS**	
**Batch**	**N**	**N controls**	**N cases**
1	319	0	0
2	709	262	0
3	954	925	0
4	287	99	0
5	607	578	0
6	352	267	0
7	1347	478	131

#### Quality control of chip data

PLINK 1.9 (24) was used for quality control of the genotype data. A pre-QC filtered SNV set (missingness <0.05) was used to exclude subjects with mismatches between reported and estimated gender and samples with call rates below a chip specific threshold. Sample call rate thresholds differed slightly between chips to account for smaller sample sizes (96–99%). After subject removal, SNV quality was assessed and variations were excluded based on the following criteria: SNV call rate <99%, deviations from Hardy-Weinberg equilibrium in controls (*p* ≤ 10^−6^) or cases (*p* ≤ 10^−10^) and SNVs with call rate differences ≥ 0.02 between cases and controls. Also, x-chromosomal markers with a haploid heterozygosity rate > 2%, missingness ≥ 0.05 or HWE *p* ≤ 10^−6^ in females were removed. A more stringent quality controlled (MAF ≥ 0.05, HWE *p* ≥ 0.05, callrate ≥ 0.99) and LD-pruned autosomal marker set was used for cryptic relatedness, heterozygosity deviation and population stratification analyses. The marker set was pruned with PLINK's indep pairwise command using *r*^2^ = 0.2, a window size of 1,500 and shifting the window 150 SNVs at each step. Additionally, several high LD regions like the extended MHC region were excluded. One subject of each pair with $\hat{\pi }$ > 0.1875 was removed, cases were generally preferred over controls. As sample contamination results in an increase of heterozygote calls and thereby an overestimation of cryptic relatedness, individuals showing a heterozygosity deviation with |*Fhet*| ≥ 0.2 were excluded. In addition, the quantity of $\hat{\pi }$ > 0.05 per individual and the distribution of $\hat{\pi }$ means were used to check for outliers and possible sample contamination. Population stratification analysis was done with EIGENSTRAT (25). SNV loadings were checked for normality and the derived principal components were used to identify and remove outlying samples.

Known duplicates on different chips were checked for concordance, keeping the sample of higher quality (i.e., sample call rate and overall chip quality). Both subjects were excluded if the concordance rate was lower than 99%.

#### Pre-phasing and imputation

Each batch was pre-phased with SHAPEIT (26) and imputed separately on the 1,000 Genomes reference panel phase 1 version 3 macGT1 (https://mathgen.stats.ox.ac.uk/impute/data_download_1000G_phase1_integrated.html) in chunks of 3 Mb using IMPUTE2 (27). Chr. X imputation was performed separately for males and females. After imputation, the seven batches were combined by retaining markers with INFO ≥ 0.6 in every batch and the combined set. Additionally, all markers with allele frequency differences > 0.1 between any of the batches were excluded. Checks for cryptic relatedness, heterozygosity deviation, and population stratification on the combined set were performed, as described before, using the same exclusion criteria on a stringent thresholded marker set (INFO > 0.8, missingness <1%, MAF > 0.05) for calling best guess genotypes with uncertainty ≤ 0.1.

#### GWAS analysis

**Figure 1:**
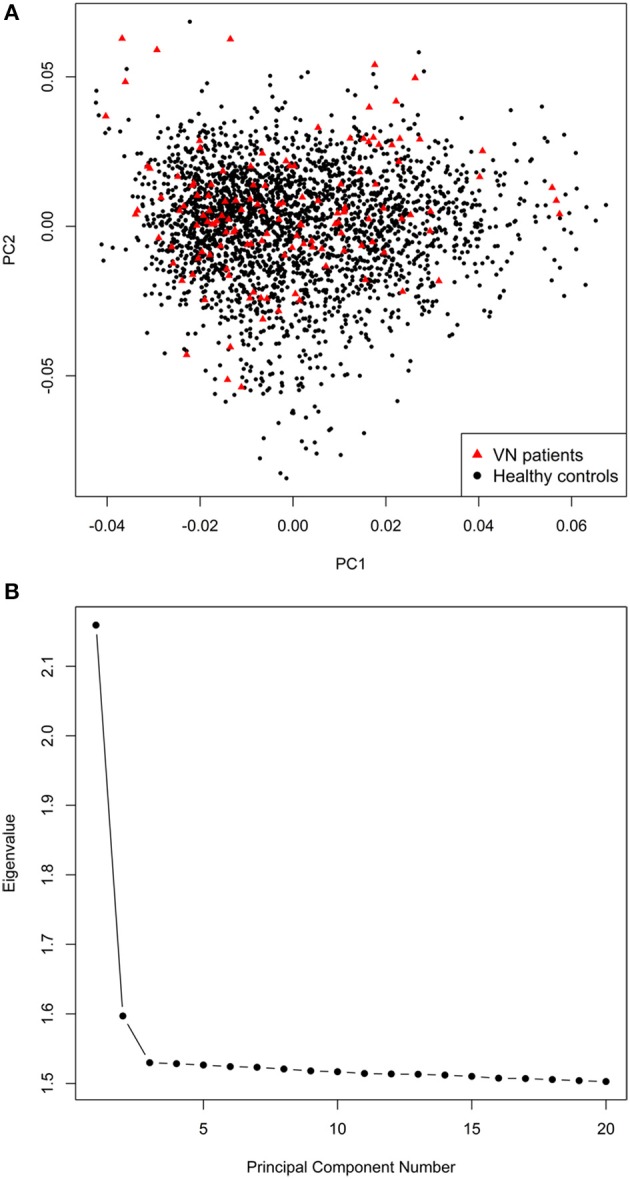
**(A)** Scatterplot of the first two principal components derived by EIGENSTRAT. **(B)** Scree-plot of principal components 1–20 derived by EIGENSTRAT.

The final mega-dataset was comprised of 4,575 individuals, including 134 VN patients and 2,618 healthy controls appropriate for association analysis spread across 6 batches (Table 1). Principal components for genome-wide association analysis were derived by EIGENSTRAT (25); 3 outlying cases and 9 controls were removed. Tracy-Widom statistics and the scree-plot of eigenvalues identified PC1 and PC2 as relevant (Figure 1). PC1 and PC2 were not associated with batch [Likelihood Ratio Test *p* = 0.15 (PC1), *p* = 0.07 (PC2)]. As VN cases were typed on one platform, controls of the six batches were used for batch effect detection. Any marker showing deviations between any of the batches was excluded (logistic regression corrected for PC1 and PC2, *p* < 0.001). After exclusion of 116,878 variants, the final dataset was comprised of 7,813,927 markers.

**Figure 2:**
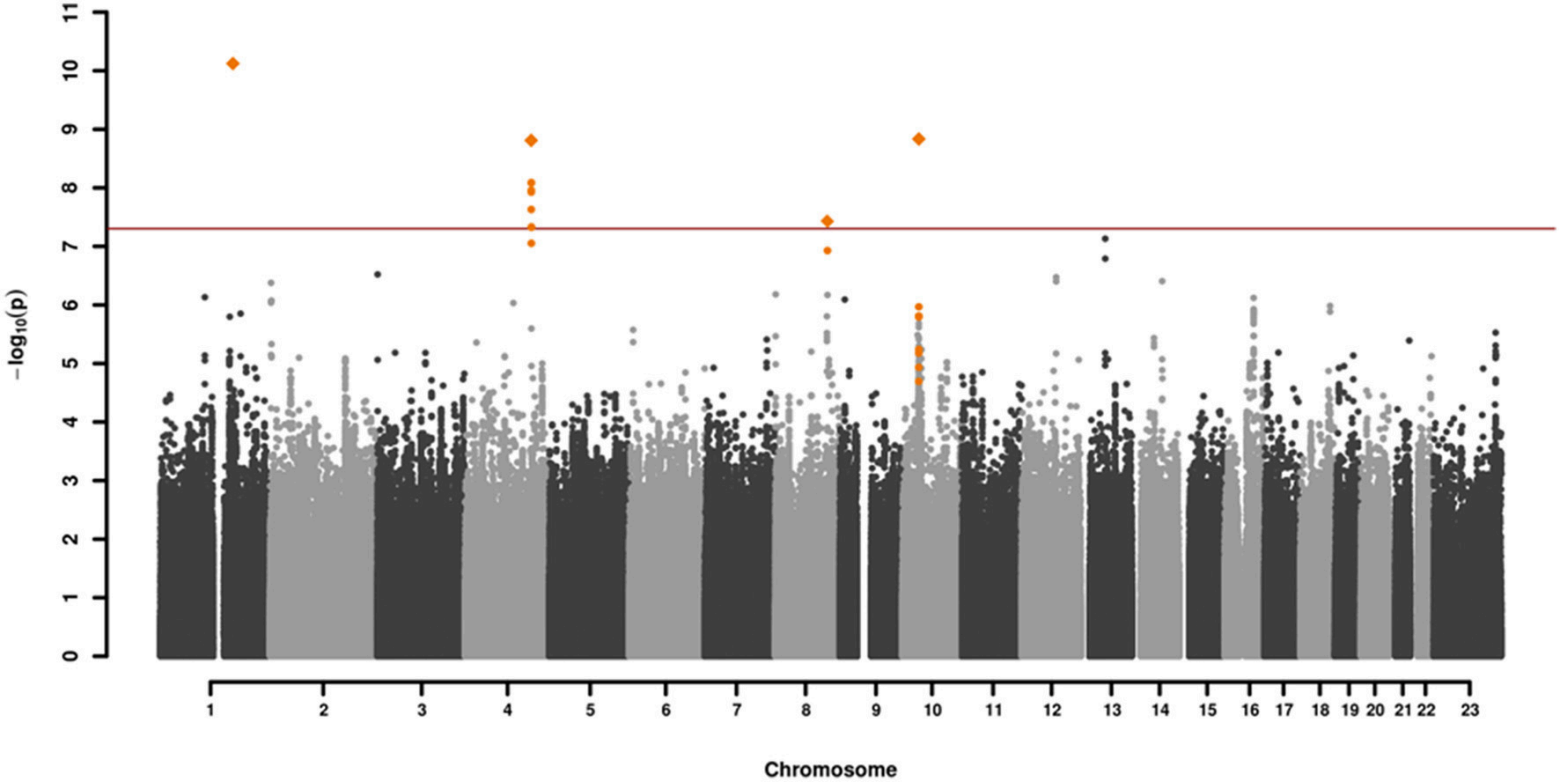
Manhattan plot of the genome-wide association analysis of 131 cases and 2,609 controls. The x-axis shows the chromosomal position, the y-axis the significance of association [–log_10_(*p*)]. The red line shows the genome-wide significance level (5 × 10^−8^). Genomic variations in orange are in LD (*r*^2^ > 0.6, *p* = 0.0001) with the index SNVs (diamonds) which represent independent genome-wide significant associations (*r*^2^ < 0.1).

**Figure 3:**
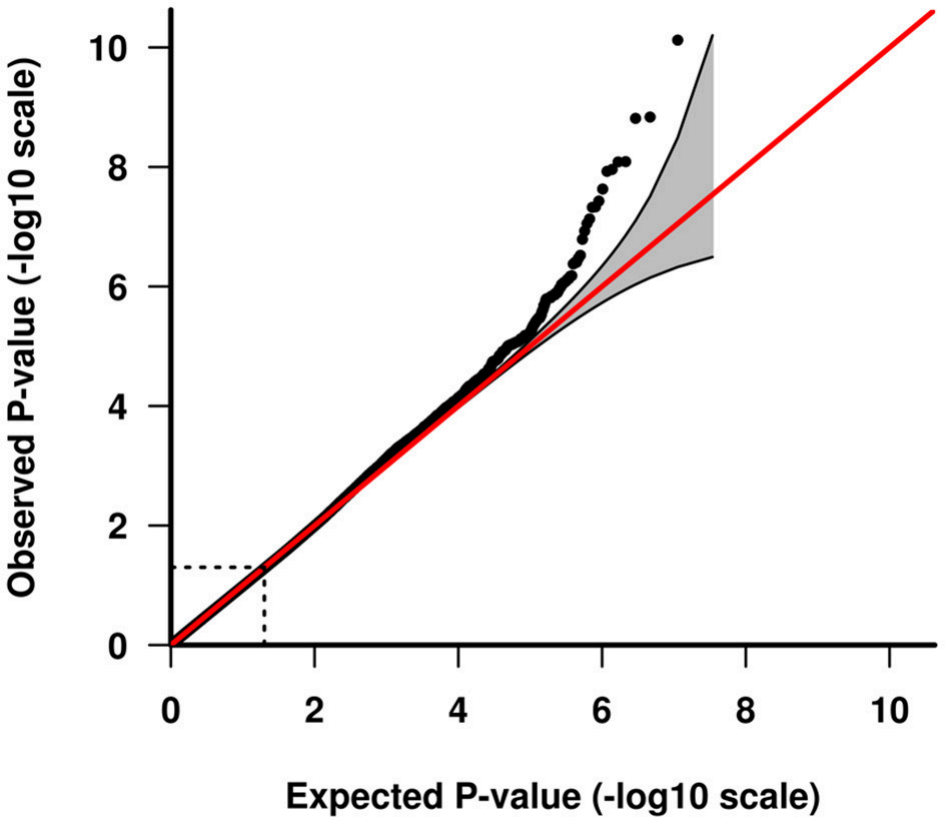
Quantile-quantile plot of GWAS analysis. The area shaded in gray indicates the 95% confidence interval under the null.

GWAS association testing of approximately 8 million variants (INFO ≥ 0.6, MAF ≥ 0.01 in cases and controls) using 131 patients with VN and 2,609 healthy subjects was conducted in PLINK 1.9 (24) applying an additive logistic regression model corrected for age, sex, PC1 and PC2. Quantile-quantile and Manhattan plots are shown (Figures 2, 3). The genomic inflation factor was 1, thus showing no sign of global inflation due to batch effects or population stratification. Results were clumped with PLINK to derive LD independent index SNVs (3,000 kb, p1 = 5 × 10^−8^, p2 = 1 × 10^−4^, *r*^2^ < 0.1).

## Results

One-hundred and thirty-one patients with VN (64 females, 51%) and 2609 healthy subjects (1402 females, 54%) were examined. The mean age (± SD) of the patients was 58.02 ± 13.6 years, mean age of controls was 47.41 ± 16.6 years. Among approximately 8 million variants tested for association we identified 9 genome-wide associated SNVs (*p* < 5 × 10^−8^) mapping to four independent loci. Hits on chromosomes 1 and 10 showed isolated signals and should therefore be interpreted with caution. The most promising association signal was found in the chromosome 4 region (leading SNV rs186544372) (Figures 2, 3, Table 2).

**Table 2:** Genome-wide association of 4 regions with vestibular neuritis.

**Chr. (region)**	**SNV**	**A1**	**Info**	**Frq_control_**	**Frq_case_**	**OR (95% CI)**	***P*-value**	**Gene (dist.)**
chr. 01 (165140414)	**rs74835610**	T	0.799	0.985	0.931	0.134 (0.073–0.245)	7.54E^−11^	LMX1A (30690)
chr. 10 (37286040–37406937)	**rs188172955**	A	0.900	0.017	0.077	5,697 (3.242–10.013)	1.46E^−9^	ANKRD30A (78196)
chr. 04 (149770763–150153801)	**rs186544372**	A	0.863	0.015	0.068	6,356 (3.488–11.583)	1.54E^−9^	NR3C2 (404324–578878)
	rs138007517	A	0.899	0.984	0.932	0,178 (0.099–0.320)	8.14E^−9^	
	rs139369934	T	0.898	0.016	0.068	5,605 (3.119–10.070)	8.23E^−9^	
	rs115679368	A	0.910	0.984	0.932	0,183 (0.102–0.327)	1.10E^−8^	
	rs186151434	C	0.912	0.984	0.932	0.184 (0.103–0.329)	1.18E^−8^	
	rs140247570	C	0.906	0.016	0.068	5.239 (2.930–9.370)	2.34E^−8^	
	rs182341058	T	0.888	0.014	0.061	5.643 (3.034–10.498)	4.65E^−8^	
	rs187446780	T	0.888	0.986	0.940	0.177 (0.095–0.330)	4.73E^−8^	
chr. 08 (118300175–118301384)	**rs1499428**	C	0.845	0.862	0.749	0.402 (0.290–0.556)	3.72E^−8^	SLC30A8 (111222) MED30 (232790)

*SNV associations are sorted by P-value. Chromosome and position denote the associated region surrounding the index SNV (in bold) containing 1 or more variation in LD (r^2^ > 0.6) with the index SNV. A1, reference allele; Frq, allele frequency; OR, odds ratio; CI, confidence interval; gene, next protein coding gene; dist., distance to index SNV in bp*.

**Figure 4:**
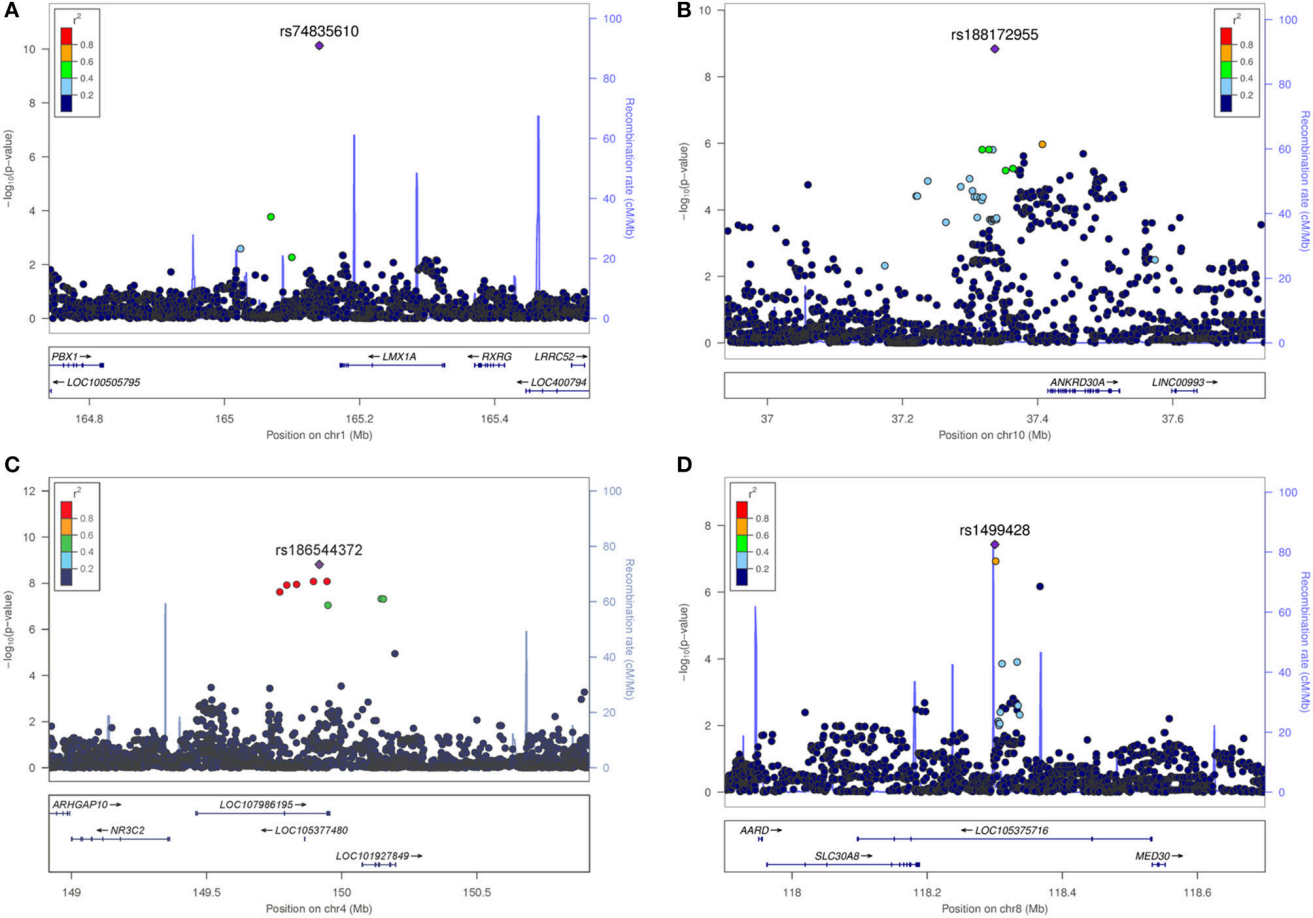
Regional association plots for the four novel vestibular neuritis loci. In order to highlight the statistical strength of the association in the context of the surrounding markers, gene annotations and estimated recombination rates (NCBI build 37) of the SNVs in the specific regions are plotted against their corresponding *P*-values (as –log_10_ values, left-hand y-axis). A purple diamond represents the SNV with the highest association signal in each locus. All other variations are represented as single dots, where dot colors indicate the LD with the associated SNV. Color coding represents the r^2^ value and respective categories are shown on the upper left hand side. Estimated recombination rates (cM/Mb) are plotted to reflect the local LD structure surrounding the associated SNV and are shown as vertical light blue lines, marked on the right-hand y-axis. Genes in the region are displayed below. Orientation of the genes is indicated by arrows. **(A)** LMX1A. Regional association plot of rs74835610 on chromosome 1q23.3. This genome-wide associated SNV (purple diamond) is localized 30 kb from LIM homeobox transcription factor 1 alpha (LMX1A). No additional variations in high LD or with low *P*-Values are present in this region. **(B)** ANKRD30A. Regional association plot of rs188172955 on chromosome 10p11.21. This genome-wide associated SNV (purple diamond) is localized 78 kb from ankyrin repeat domain 30A (ANKRD30A). Additional variations in LD (r^2^ between 0.2 and 0.8) surround this SNV but fail to reach genome-wide significance. **(C)** NR3C2. Regional association plot of rs186544372 on chromosome 4q31.23. The most significant SNV in this region as well as five other genome-wide associated SNVs in high LD (*r*^2^ > 0.8, red dots) are localized on a predicted long ncRNA (LOC107986195). The next coding gene, nuclear receptor subfamily 3 group C member 2 gene (NR3C2) is localized in 100 kb distance. **(D)** SLC30A8/MED30. Regional association plot of rs1499428 on chromosome 8q24.11. This genome-wide associated SNV (purple diamond) is localized in the intronic regions of two overlapping ncRNAs (LOC105375717, LOC105375716). The next coding genes are solute carrier family 30 member 8 (SLC30A8) and mediator complex 30 (MED30).

The most significant SNV rs74835610 (*p* = 5.54 × 10^−11^) in this study was localized on chromosome 1q23.3 app. 30 kb downstream to LIM homeobox transcription factor 1 alpha (LMX1A), a gene coding for a transcription factor, involved in the regulation of insulin gene transcription (28) as well as in the development of dopamine producing neurons during embryogenesis (29) (Figure 4A).

Next in line was rs188172955 (*p* = 1.46 × 10^−9^) on chromosome 10p11.21 which is localized 78 kb distant to the ankyrin repeat domain 30A gene (ANKRD30A, NY-BR-1), a DNA-binding transcription factor primarily expressed in mammary epithelium and testis (30) (Figure 4B). The region on chromosome 4q31.23, represented by the index SNV rs186544372 (*p* = 1.54 × 10^−9^) contains 7 additional genome-wide associated SNVs, with 5 localized in the predicted long non-coding RNA (ncRNA) LOC107986195, spanning app. 500 kb (rs138007517, *p* = 8.14 × 10^−9^; rs139369934, *p* = 8,23 × 10^−9^; rs115679368, *p* = 1.10 × 10^−8^; rs186151434, *p* = 1.18 × 10^−8^; rs140247570, *p* = 2.34 × 10^−8^) and an additional 2 localized in the long intergenic non-protein coding RNA 2355 (rs182341058, *p* = 4.65 × 10^−8^, rs187446780, *p* = 4.73 × 10^−8^). The next coding gene, nuclear receptor subfamily 3 group C member 2 (NR3C2), is app. 400 kb distant and codes for the mineralocorticoid receptor (MC-R), which is activated by mineralocorticoids such as aldosterone, thereby influencing the salt and water balance within a variety of target cells (31) (Figure 4C).

The region on chromosome 8q24.11 with the index SNV rs1499428 (*p* = 3.72 × 10^−8^) is localized in an intronic region of 2 overlapping ncRNAs (LOC105375717, LOC105375716), 111 kb downstream of solute carrier family 30 member 8 (SLC30A8) and also 232 kb upstream of mediator complex 30 (MED30) (Figure 4D). SLC30A8 encodes the zinc efflux transporter ZnT8 which is involved in the accumulation of zinc in intracellular vesicles, MED30 is part of the mediator complex, a multiprotein complex required for gene transcription by RNA polymerase II (32).

None of the significant associated variants could be directly assigned to a protein coding gene. The minimal distance to the nearest gene was app. 30 kb (LMX1A, rs74835610), maximum distance app. 400 kb (NR3C2, rs186544372).

## Discussion

In this study a genome-wide association with VN was found for four regions. The next protein coding genes in those regions can be divided into two functionally different groups: virus hypothesis and insulin metabolism.

The first group contains three hits on chromosomes 4q31.23 (NR3C2), 8q24.11 (MED30), and 10p11.21 (ANKRD30A) that show a connection to the virus hypothesis. Two hits are localized in predicted ncRNAs, linking the adjacent genes NR3C2 (host factor for HSV-1) and MED30 (HIV-1 replication) to VN, the third hit is localized near ANKRD30A (host factor for HIV-1).

The second group consists of LMX1A (1q23.3) and SLC30A8 (8q24.11), which are involved in insulin metabolism.

### Group 1: virus hypothesis

The predicted long ncRNA (LOC107986195) on chromosome 4q31.23 provides a physical link to NR3C2, encoding the mineralocorticoid receptor (MC-R) which together with mineralcorticoids regulates ion-homeostasis mainly in epithelial cells. In addition to acting as a ligand-dependent transcription factor which by binding to mineralocorticoid response elements leads to a late response following the transactivation of specific target genes, MC-R seems to directly transactivate unrelated receptors, thereby influencing intracellular signaling pathways more rapidly (33).

Also, NR3C2 was shown to be a host factor for HSV-1 replication. After knock down via siRNA depletion, NR3C2 specifically enhanced HSV-1 replication (34). Furthermore, using a murine model of HSV-1 infection a role of the MC-R in the neuroendocrine-mediated modulation of antiviral immunity has been discussed (35). For stress-induced glucocorticoids a strong suppression of CD8^+^ T cell immune responses could be demonstrated, leading to diminished antiviral immunity (36). This together with the implication of an intricate interdependence of glucocorticoid receptors (GC-R) and MC-R functions points toward a participation of both in antiviral immune response.

Apart from mineralocorticoids, the human MC-R has a high affinity for glucocorticoids which, in the form of synthetic glucocorticoids like methylprednisolone, are used in the therapy of vestibular neuritis as well as other vertigo diseases (37, 38). The therapeutic effect of glucocorticoid application seems to be dependent not only on tissue type and local concentration but also on a concerted action of GC-R and MC-R on transcriptionally and non-transcriptionally mediated signaling pathways (39).

Another hit is localized on chromosome 8q24.11 in the intronic regions of two overlapping ncRNAs (LOC105375717, LOC105375716), thereby providing a link to the neighboring genes SLC30A8 and MED30.

MED30 is part of the tail of the mediator complex, a multiprotein complex required for gene transcription by RNA polymerase II, thereby controlling the expression of genes through subunit specific interaction, epigenetic regulation, elongation and termination of transcription, processing of mRNA, activation of noncoding RNA as well as formation of super enhancers (32). MED30 has been shown to be involved in replication of HIV-1, a knockdown leading to impaired viral replication (40). Although not directly linked to HSV-1 itself, several other genes of the mediator complex have been shown to be host factors for HSV-1 infection. For example, MED23 has been shown to conduct a HSV-1 specific anti-viral effect. Interaction of the transcription factor IRF7 with MED23 induced an upregulated expression of the interferon lambda family. Interferon lambda, on the other hand, has been suggested to play a crucial role in HSV-1 immune control (34). Also, Tat-mediated transcription was impaired after RNAi knockdown (41). Functional roles of MED30 are poorly understood. It was found to play a fundamental role in the regulation of function and integrity of mitochondria in mice homozygous for a missense mutation (42).

The variants attributed to MED30 and NR3C2 are localized in predicted ncRNAs with unknown function, but involvement in antiviral host response has recently been shown for this type of RNA (43). Therefore, it could be hypothesized that the associated ncRNAs exert a potentially regulatory influence on the adjacent genes.

Another link to virus response is provided by a hit on chromosome 10p11.21, next to the ANKRD30A/NY-BR-1 gene. Though mostly known as an antigen associated with breast cancer progression (30), this gene was also determined to be an HIV-1 host susceptibility factor using whole-genome RNA interference screens (41), and shows a strong signature of positive virus-driven selection in an evolutionary analysis (44). Further work is necessary to characterize the role of ANKRD30A regarding its involvement in HIV-1 biology, especially as ANKRD30A has been shown to be one of very few candidates with potential as a drug target (45).

### Group 2: insulin metabolism

The most significant SNV rs74835610 is localized 30 kb from the transcription factor LMX1A. LMX1A and LMX1B play an essential role during the development of dopaminergic progenitors in the midbrain. In a mouse model Lmx1a was shown to be essential for the conversion of non-neuronal floor-plate cells into neuronal dopamine progenitors (29). Lmx1a-deficiency in mice reduced Tph1 expression, the rate-limiting enzyme for serotonin biosynthesis (46). In addition to its role in the development of dopamine producing neurons during embryogenesis (29) and serotonin biosynthesis (46), Lmx1A also seems to have a role in the positive regulation of insulin gene transcription (28).

The associated variation localized on 8q24.11 could be attributed to MED30 as well as to SLC30A8. The human SLC30 gene family of solute carriers seems to be involved in the maintenance of zinc homeostasis and in facilitation of zinc transport from the cytoplasm into specialized intracellular compartments or the extracellular space (47). SLC30A8 encodes the zinc efflux transporter ZnT8 which affects the accumulation of zinc in intracellular vesicles and shows high expression levels in the islets of Langerhans cells of the pancreas. Zinc is required for zinc-insulin crystallization in the immature granules of these cells. By stimulating these cells with glucose, large amounts of the mature granules are secreted to the extracellular space, thereby delivering zinc together with insulin. It has been suggested that zinc plays a role in the paracrine and/or autocrine signaling mechanisms in islet cells (48). Loss-of-function mutations in SLC30A8 seems to be strongly protective against type 2 diabetes (49).

In summary, we identified four regions attributable to five genes, three of which (NR3C2, ANKRD30A, MED30) are involved in viral processes: NR3C2 was shown to be a host factor for HSV-1 replication, ANKRD30A is a host factor for HIV-1 infection and MED30 has been shown to be involved in replication of HIV-1.

The second set of genes comprising LMX1A and SLC30A8 is involved in insulin metabolism and might be relevant for the frequently occurring metabolic comorbidities like T2DM, supporting the hypothesis of susceptibility to insulin resistance as a consequence of an increased exposure to pathogens leading to chronic low-grade inflammation.

Though the number of VN cases is very small for a GWAS analysis, and the isolated association signals on chromosomes 1 and 10 should be considered with caution, these findings—in addition to other studies using different methods—provide preliminary support for the view that viral inflammation is a causative factor in the etiology of VN.

## Limitations

The study is limited by the small sample size and the fact that individuals were genotyped on several platforms. Minor allele frequencies of genome-wide significant markers are in general low and larger sample sizes and independent replications are required to explore the causative factors of VN.

## Author contributions

DR, MS, and IG were involved in the conception and design of study. MH and AH participated in the acquisition and analysis of the data. BK was responsible for the statistical analysis. All authors contributed substantially to the manuscript.

### Conflict of interest statement

MS is Joint Chief Editor of the Journal of Neurology, Editor in Chief of Frontiers of Neuro-otology and Section Editor of F1000. He has received speaker's honoraria from Abbott, Actelion, Auris Medical, Biogen, Eisai, GSK, Henning Pharma, Interacoustics, MSD, Otometrics, Pierre-Fabre, TEVA, UCB. He acts as a consultant for Abbott, Actelion, Heel, IntraBio, and Sensorion. The remaining authors declare that the research was conducted in the absence of any commercial or financial relationships that could be construed as a potential conflict of interest. The reviewer MM-B and handling Editor declared their shared affiliation.
